# Sesamin protects against neurotoxicity via inhibition of microglial activation under high glucose circumstances through modulating p38 and JNK signaling pathways

**DOI:** 10.1038/s41598-022-15411-3

**Published:** 2022-07-04

**Authors:** Prachya Kongtawelert, Chayanut Kaewmool, Thanyaluck Phitak, Mattabhorn Phimphilai, Peraphan Pothacharoen, Thuzar Hla Shwe

**Affiliations:** 1 Thailand Excellence Center for Tissue Engineering and Stem Cells, Department of Biochemistry, Faculty of Medicine, Chiang Mai University, Chiang Mai, 50200 Thailand; 2 Division of Endocrinology, Department of Internal Medicine, Chiang Mai University, Chiang Mai, Thailand

**Keywords:** Biochemistry, Neuroscience

## Abstract

Diabetes mellitus (DM), one of the principal causes of morbidity and mortality worldwide, is implicated in the progression of age-related neurodegenerative diseases (NDDs), in which microglial activation is a crucial mediator. Sesamin, a kind of phytochemical, shows inhibitory effects on microglial activation. The present study studied whether sesamin protects against neurotoxicity triggered by high glucose-induced microglial activation. We firstly demonstrated that high doses of glucose, which mimics hyperglycemia in DM, did induce the activation of murine BV2 microglial cells, increasing inflammatory responses such as the production of ROS or inflammatory mediators like IL-1β, TNF-⍺, and nitric oxide, through activation of p38 and JNK signaling pathways. Next, conditioned medium (CM) collected from high glucose-activated BV2 cell culture was used to show aggravated neurotoxicity in differentiated PC12 cells, indicating that high glucose-activated microglia could induce neurotoxicity. Interestingly, pretreatment of BV2 cells with sesamin diminished high glucose-induced microglia activation and inflammatory responses. Moreover, neurotoxicity in PC12 cells was found to be decreased in the group treated with CM from the sesamin-pretreated BV2 cell culture, suggesting sesamin inhibited microglial activation, thereby protecting neurons from activated microglia-mediated neurotoxicity. Thus, sesamin might be a potential compound to use in the prevention of diabetic-induced NDDs.

## Introduction

Diabetes mellitus (DM) is a persistent metabolic disease characterized by chronic hyperglycemia with tissue damage-associated complications, which are leading causes of mortality globally^1,2^. Neuropathies including neurodegenerative diseases (NDDs) are serious complications commonly found in diabetic patients^3^ and accumulated verifications demonstrated that diabetes was significantly involved in developing NDDs^4,5^. NDDs are normally characterized by progressive destruction of neurons in the central nervous system (CNS), resulting in dysfunctions of specific brain areas, which lead to the loss of memory, cognitive decline, and movement problem; and Alzheimer's disease (AD) and Parkinson's disease (PD) are most commonly found members in the group of NDDs^6,7^.

Type 2 DM (T2DM), insulin-resistant diabetes, is the most prevalent type of diabetes connected with NDDs^8,9^. Patients with T2DM can heighten the risk of Alzheimer’s disease (AD) and Parkinson’s disease by about 1.5 and 2.2-fold, respectively, compared with patients without T2DM^10,11^. Recently, an abundance of research has investigated the relationship between DM and NDDs; and neuroinflammation is found to be a critical link between them. Persistent hyperglycemia of diabetic state can initiate oxidative stress and inflammation in the brain, consequently resulting in neurodegeneration^12–15^.

The overactivation of microglia, the CNS macrophages, is known to be a major mediator of inflammation-induced neurodegeneration. Resting microglia can be activated by various pathological stimuli in neurological environment as a physiological response to maintain brain homeostasis. Nevertheless, inordinate microglial activation generates inflammatory mediators and ROS excessively resulting in neuroinflammation and oxidative stress, two prerequisites for development of neuronal injuries which can ultimately lead to neurodegenerative disorders^16–18^. Previous in vivo studies showed that hyperglycemia in diabetic model can trigger microglial activation-mediated neuroinflammation and this process implicates in neurodegenerative conditions such as dementia, AD, and PD^15,19,20^.

Previous research demonstrated that brain glucose level is increased in diabetic hyperglycemic rats^20,21^. Accordingly, the treatment with high glucose concentration has been widely used to mimic diabetes-associated hyperglycemic environment in several in vitro studies that determine mechanisms of hyperglycemia-induced neuropathies. High glucose can trigger the activation of microglia, resulting in the production of reactive oxygen species (ROS) and inflammatory mediators such as nitric oxide (NO), tumor necrosis factor (TNF)-⍺, and interleukin (IL)-1β. The mitogen‑activated protein kinase (MAPK) signaling pathways, such as p38, JNK and ERK, were reported to be responsible for the glucose induced activation of microglial cells^22–24^. Taken together, inhibition of microglial activation and inflammation triggered by hyperglycemia might be a therapeutic approach for the prevention of neurological complications, especially NDDs, in DM.

Because of lower side effects, various phytochemicals are increasingly investigated for their advantageous effects on health^25^. Sesamin, a bioactive lignan compound found in sesame seed oil (*Sesamum indicum*), is one of the popular phytochemicals that have been determined for its biological abilities. Sesamin was reported to have potent antioxidant and anti-inflammatory activities, correlating with its various beneficial effects on multiple diseases. Prior studies revealed that sesamin exhibits therapeutic benefits on DM, complications of DM, and NDDs^26–31^ while it was shown to inhibit microglial activation and consequent neuroinflammation in addition^32,33^. Yaghoob Farbood and colleagues found that sesamin can protect against diabetes-associated cognitive decline in rats through the reduction of oxidative stress and neuronal death^34^. However, the effect of sesamin on neurotoxicity, particularly the microglial activation-mediated neurotoxicity initiated by hyperglycemia, is still not completely comprehended. Hence, in our study, we examined whether sesamin restrains high glucose-induced microglial activation and thereby defends PC12 cells from microglial activation-induced neurotoxicity.

## Results

### Cytotoxicity of glucose and sesamin on BV2 cells

**Figure 1:**
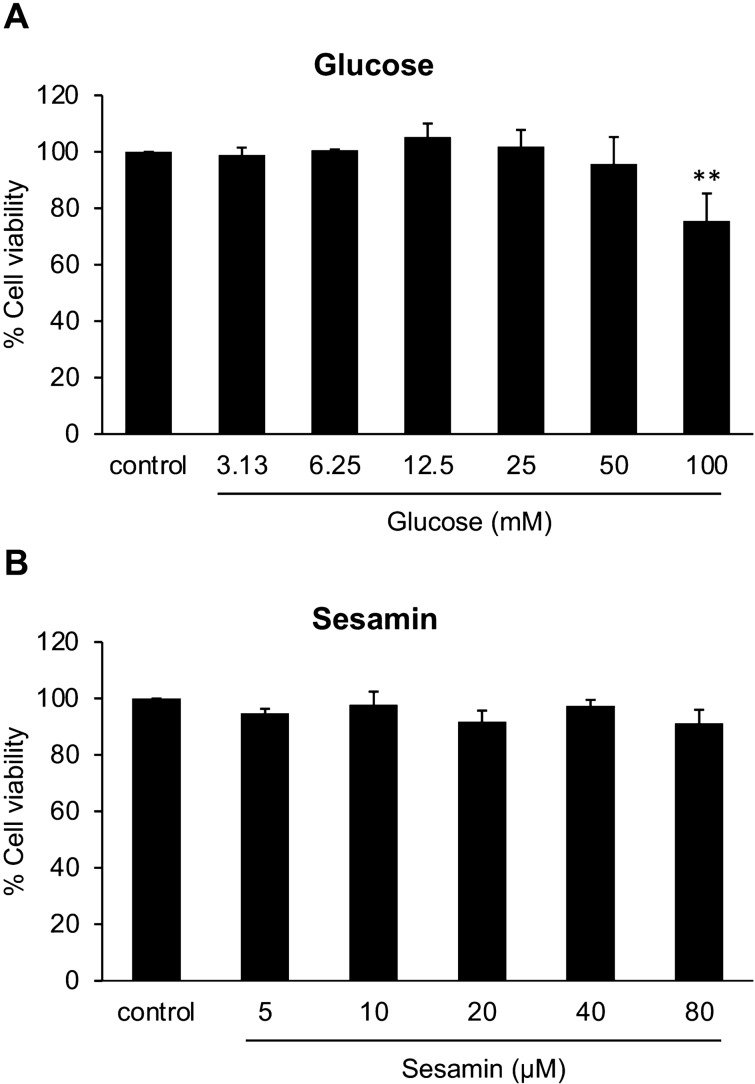
Effects of (**A**) glucose and (**B**) sesamin (Se) on BV2 cell viability. Cells were incubated with diverse doses of glucose (0–100 mM) or sesamin (0–80 μM) for 48 h. The BV2 cell viability was detected by MTT assay. The results are showed as the mean ± SEM from three independent experiments. **P < 0.05 versus the control group.

In our study, high glucose concentrations, which mimic the in vitro glucose levels of diabetes and its complications, were utilized to stimulate microglial activation. It is essential to select a non-cytotoxic concentration of glucose for BV2 cells. Therefore, MTT assay was proceeded to determine the cytotoxic effect of glucose on BV2 cells. The cells were treated with different concentrations (0–100 mM) of glucose for 48 h. We found that concentrations of glucose up to 50 mM had no cytotoxicity on BV2 cells, while 100 mM of glucose showed a significant cytotoxicity (Fig. 1A). According to this result, 12.5, 25, and 50 mM glucose concentrations were chosen for further investigations.

To make certain that sesamin concentrations used in the study were not toxic to BV2 cells, the cells were incubated with sesamin at the concentrations of 0–80 μM (twofold dilution). After 48 h of incubation, all doses of sesamin did not reveal any noticeable cytotoxicity (Fig. 1B). Therefore, three concentrations of sesamin, 10, 20, and 40 μM, were utilized in the next experiments.

### High glucose enhanced microglial activation in BV2 cells

Previous studies have proved that high glucose can trigger inflammatory activation of BV2 microglial cells. In the activated state, these cells were transformed from the resting ramified phenotype to the activated amoeboid phenotype. High glucose-associated activated BV2 cells could produce ROS and inflammatory-mediated molecules, such as NO, TNF-⍺, IL-1β, and IL-6. These ROS and inflammatory mediators promote oxidative stress and neuroinflammation, which consequently cause neuronal damage or death^22,35–37^.

**Figure 2:**
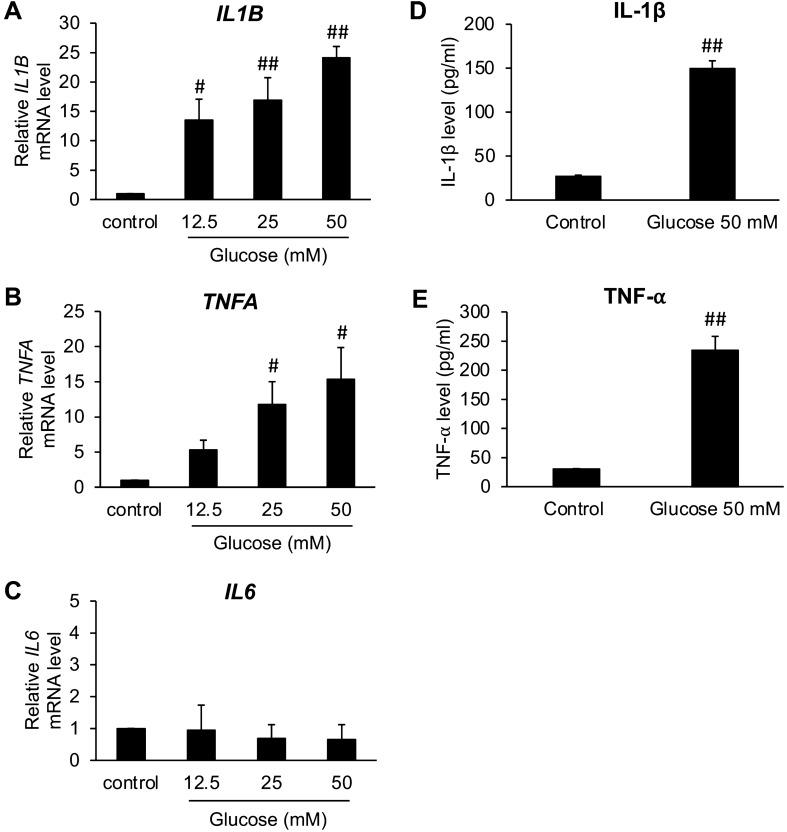
Effects of high glucose on pro-inflammatory cytokines, IL-1β, TNF-⍺, and IL6, gene expression (**A**), and secretion (**B**) in BV2 cells. Cells were incubated with either three doses of glucose (12.5, 25, 50 mM) for 24 h or only 50 mM glucose for 48 h. Next, the level of mRNA and protein of these pro-inflammatory cytokines were measured by real time-PCR and ELISA, respectively. The data are displayed as the mean ± SEM from three individual experiments. ^#^*P* < 0.05 or ^##^*P* < 0.01 versus the control group.

To confirm that high glucose could induce microglial activation, BV2 cells were stimulated by glucose at high concentrations (12.5, 25, and 50 mM). We firstly conducted Real-time PCR to examine the mRNA level of pro-inflammatory cytokines, which are IL-1β, TNF-⍺, and IL-6. As shown in Fig. 2A–C, glucose doses at 25 and 50 mM significantly elevated the mRNA level of all cytokines in BV2 cells, except IL-6. Accordingly, glucose dose at 50 mM was selected to investigate whether high glucose dose can augment the excretion of IL-1β and TNF-⍺ from the BV2 cells into the culture medium. From the result of ELISA (Fig. 2D,E), we discovered that 50 mM glucose could significantly augment the release of IL-1β and TNF-⍺ from BV2 cells. All these data indicated that high glucose (50 mM) does upregulate the production of IL-1β and TNF-⍺ at the transcriptional level.

**Figure 3:**
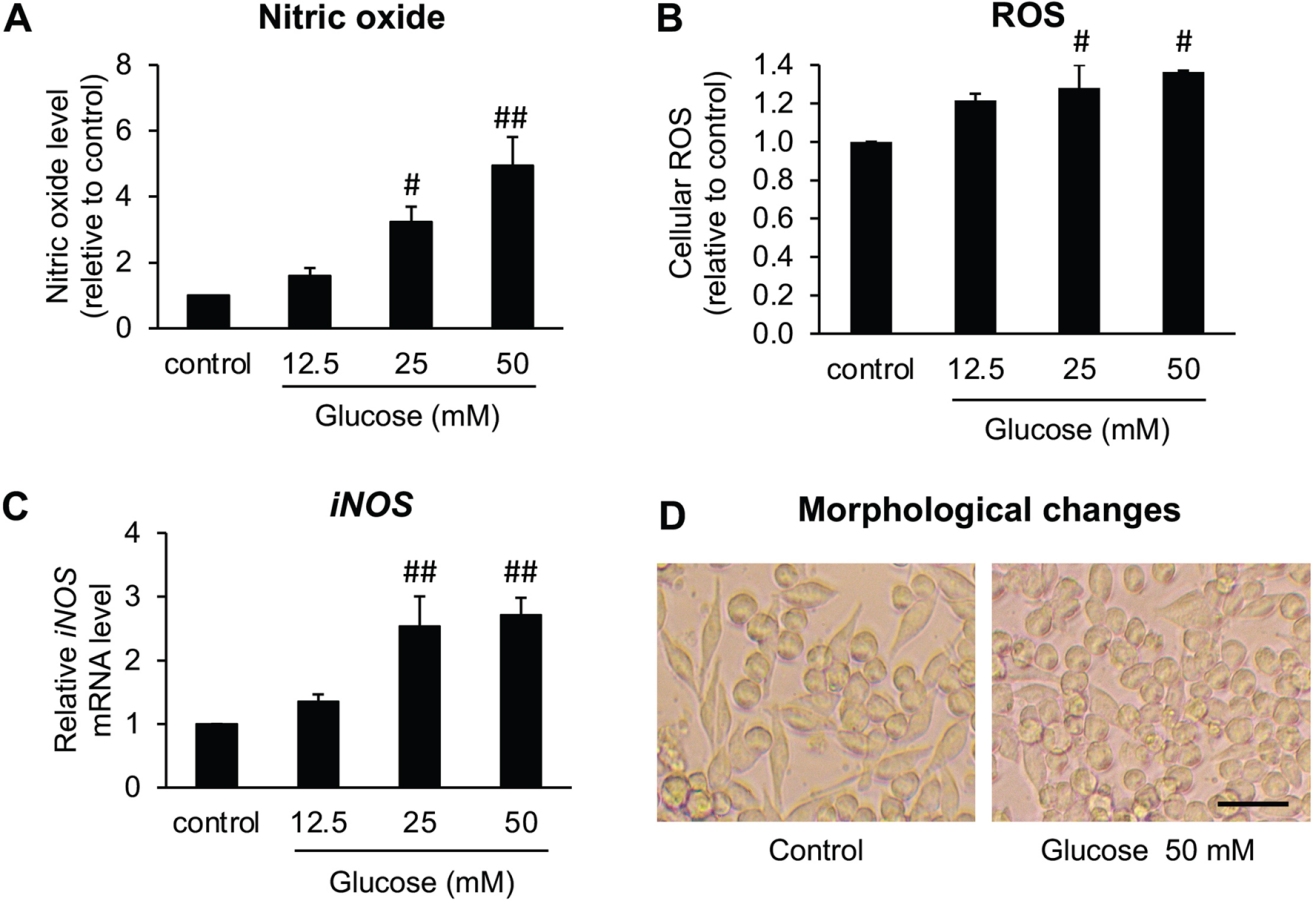
Effects of high glucose on features indicating microglial activation in BV2 cells, including nitric oxide (NO) release (**A**), cellular ROS generation (**B**), *iNOS* gene expression (**C**), and morphological alteration (**D**). Cells were incubated with either three doses of glucose (12.5, 25, 50 mM) or only 50 mM glucose for 24 or 48 h depending on the experiment. The experiments, composed of Griess’s assay, H2DCFDA assay, real-time PCR, and the observation under a light microscope, were performed to determine the level of nitric oxide, cellular ROS, *iNOS* mRNA, and also the morphological alteration, respectively. In morphological observation, photos were taken at ×20 magnification and selected from one experiment as a representative from three independent experiments. The scale bar is 50 μm. The values are demonstrated as the mean ± SEM from three independent experiments. ^#^*P* < 0.05 or ^##^*P* < 0.01 versus the control group.

Free radicals generated by activated microglia, including NO and ROS, play a key role in neurodegenerative progression^18^. Thus, we next studied the effects of high glucose in BV2 cells on the release of NO, ROS, and the gene expression of inducible nitric oxide synthases (*iNOS*), which is the inducible enzyme responsible for NO production, using Griess’s assay, DCFDA assay, and real-time PCR respectively. After exposing BV2 cells to high glucose doses (25 and 50 mM), significant increases in cellular ROS production (Fig. 3B), NO release and *iNOS* gene expression, were found out, reflecting the induction of microglial activation by high glucose (Fig. 3A,C).

The alteration of BV2 cells’ morphology has been used to affirm the activation of microglia^38,39^. In this study, shifting to the activated form of BV2 microglial cells was observed under the light microscope after they were treated with 50 mM glucose for 48 h. The morphology of untreated BV2 cells displayed a slender soma with the distal ramification, being characterized as the resting ramified microglia. Following stimulation with high glucose, BV2 microglia had a larger spherical soma with shorter branches, and this morphology was characterized as the activated amoeboid microglia (Fig. 3D). Altogether, we could summarize that high glucose could induce the inflammatory microglial activation and consecutive production of neurotoxic molecules such as IL-1β, TNF-⍺, NO, and ROS. Glucose at 50 mM was applied to the later experiments because of its highest potency to promote inflammatory responses in BV2 cells.

### High glucose-enhanced microglial activation is mediated by the activation of p38 and JNK of MAPK signaling pathways

**Figure 4:**
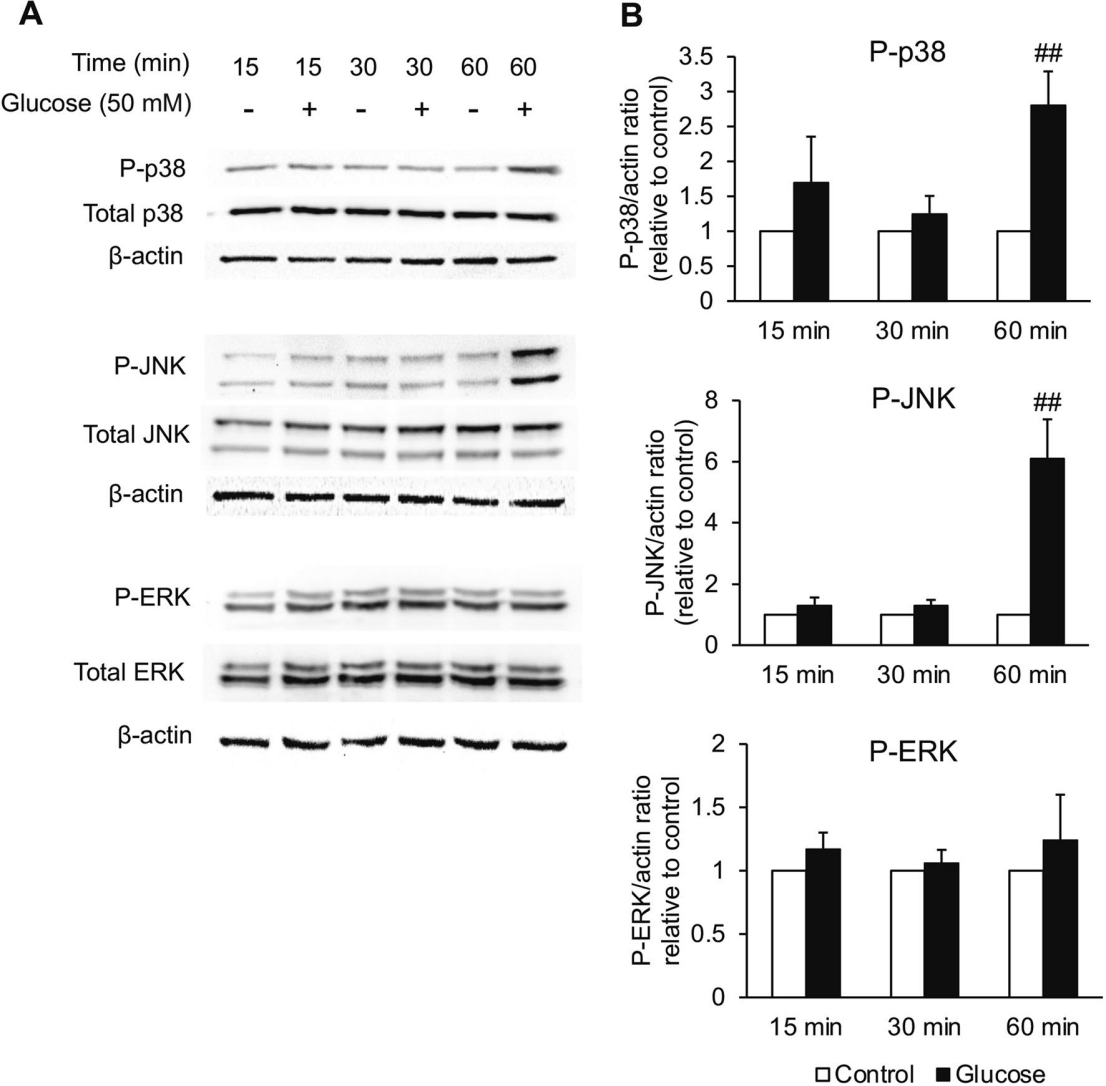
Effects of high glucose on the activation of MAPKs signaling pathways in BV2 cells. The cells were incubated with 50 mM of glucose for different times, such as 15, 30, and 60 min. The protein expression of phosphorylated(P)-p38, P-JNK, P-ERK, p38, JNK, and ERK was examined by western blot analysis (**A**). The band intensity of these proteins was quantified by Total lab TL120 and was utilized to analyze the level of protein expression by normalizing with the band intensity of β-actin (**B**). Data are presented as the means ± SEM from three distinct experiments. ^#^*P* < 0.05 or ^##^*P* < 0.01 versus the control group.

Activation of MAPK signaling pathways, comprising p38, JNK, and ERK, has recently been reported to be a crucial process correlated to hyperglycemia-motivated inflammatory reactions in activated microglia^23,39^. Therefore, the phosphorylation of MAPKs in BV2 cells was explored by western blot analysis following the high glucose treatment. We found that after stimulation with 50 mM glucose for 60 min, the phosphorylated forms of p38 and JNK were increased significantly. However, the significant upregulation was not observed in ERK signaling pathway (Fig. 4A,B), indicating that high glucose triggered microglial activation via p38 and JNK signaling pathways.

### Sesamin restrained high glucose-persuaded microglial activation

**Figure 5:**
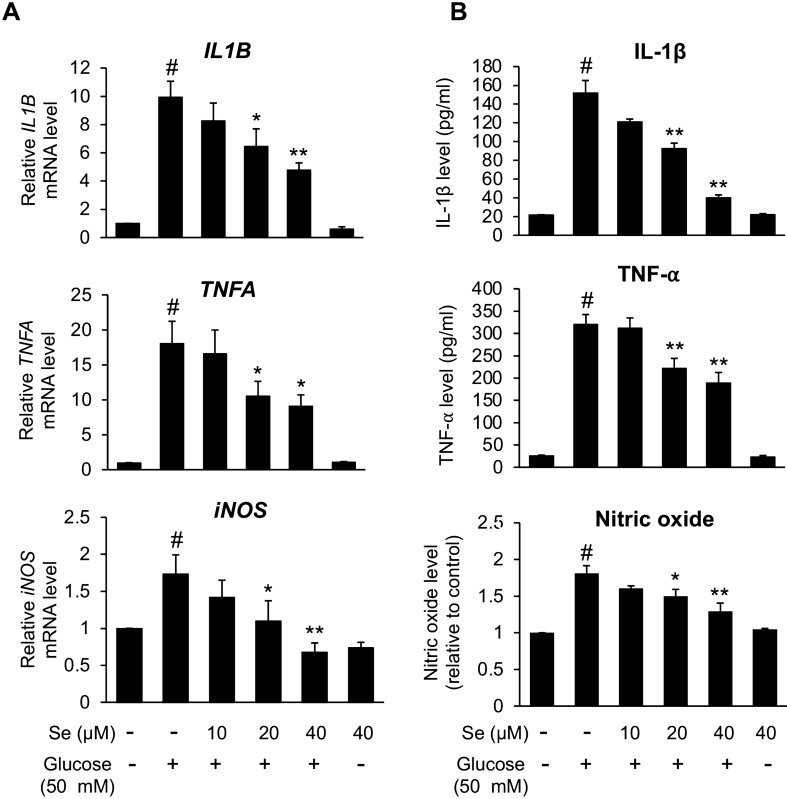
Effects of sesamin on inflammatory gene expression (**A**) and secretion of inflammatory mediators (**B**) in high glucose-activated BV2 cells. The pretreatment of BV2 cells with sesamin (10, 20, 40 μM) for 4 h was performed before BV2 cells were treated with 50 mM of glucose. Following the incubation for 24 or 48 h, the gene expression (*IL1B*, *TNFA*, *iNOS*) and the secretion of inflammatory mediators (IL-1β, TNF-⍺, NO) were tested by real-time PCR and ELISA, other than NO that was studied by Griess’s assay. The outcomes are shown as the means ± SEM from three individual experiments. ^#^*P* < 0.05 versus the control group and *P < 0.05 or **P < 0.01 versus the glucose-treated group.

**Figure 6:**
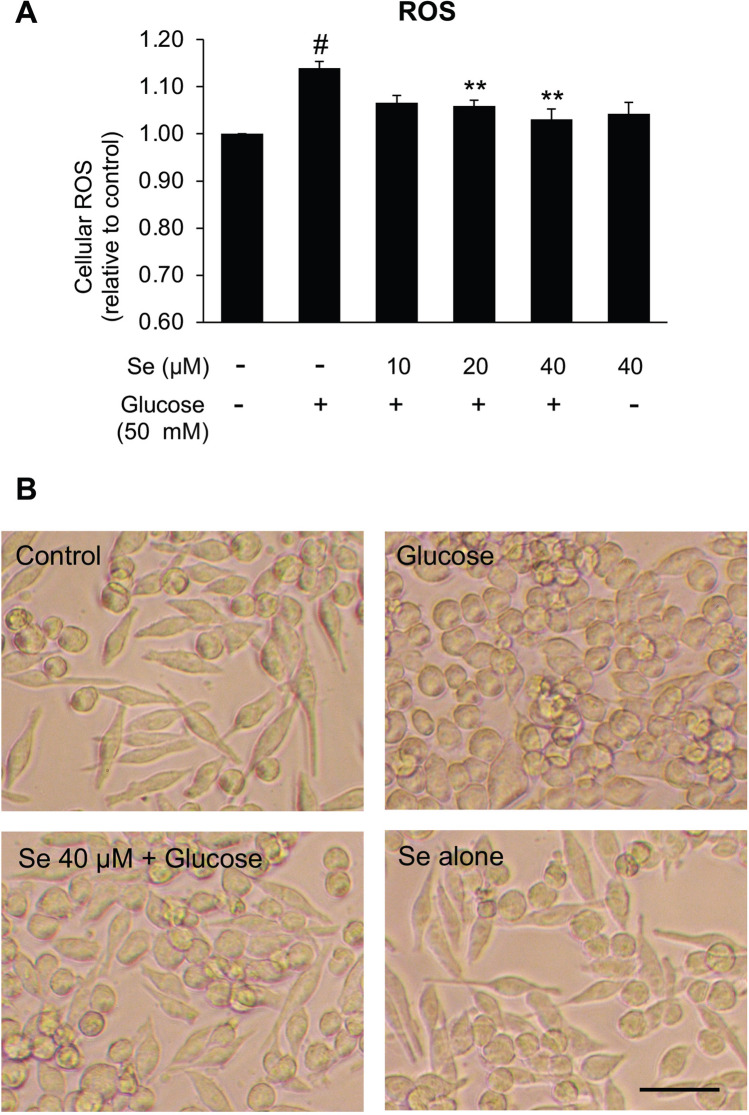
Effects of sesamin on cellular ROS generation (**A**) and morphological alteration (**B**) in high glucose-activated BV2 cells. The pre-incubation of BV2 cells with sesamin (10, 20, 40 μM) for 4 h was carried out before BV2 cells were incubated with 50 mM of glucose. After 24 or 48 h, cellular ROS generation and morphological change were inquired by H2DCFDA assay and the observation under a light microscope), respectively. In morphological observation, photos were taken at ×20 magnification and selected from one experiment as a representative from three independent experiments. The scale bar is 50 μm. The results are exhibited as the means ± SEM from three separate experiments. ^#^*P* < 0.05 versus the control group and *P < 0.05 or **P < 0.01 versus the glucose-treated group.

To examine the effect of sesamin on high glucose-induced microglial activation, BV2 cells were pre-incubated with sesamin at 10, 20, and 40 μM before high glucose (50 mM) was added to the media. In the absence of pretreatment with sesamin, 50 mM glucose obviously caused microglial activation since the results showed increased production of neurotoxic mediators (IL-1β, TNF-⍺, NO, ROS), enhanced expression of their genes (*IL1B*, *TNFA*, and *iNOS*), and the transformation to an activated form morphologically (Figs. 5, 6). In comparison with the sesamin untreated group, we could see that pre-incubation with sesamin at 20 and 40 μM significantly reduced the level of inflammatory mediators (IL-1β, TNF-⍺, NO) released by high glucose-activated BV2 microglia (Fig. 5B), and the expression of the indicated genes (Fig. 5A). These inhibitory effects of sesamin were dose-dependent too. Moreover, pretreating with sesamin could substantially reverse the high glucose-elevated ROS production and morphological changes in BV2 cells (Fig. 6A,B). From the results, we could suggest that sesamin inhibited the activation of microglia induced by high glucose and consequent inflammatory responses.

### Sesamin diminished high glucose-induced microglial activation by modulating the activation of p38 and JNK signaling pathways

**Figure 7:**
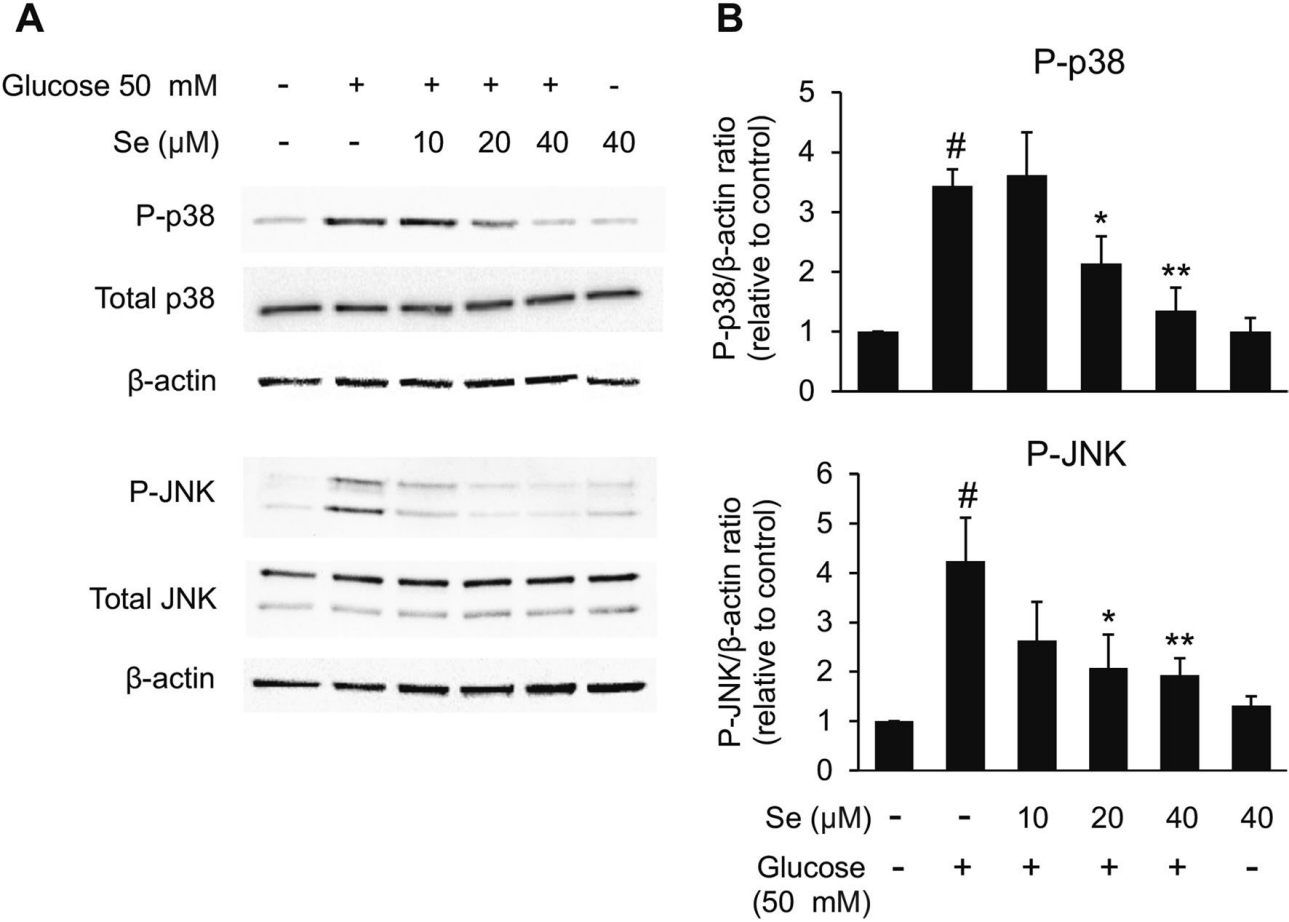
Effects of sesamin on the activation of p38 and JNK MAPKs signaling pathways in high glucose-activated BV2 cells. The cells were pretreated with sesamin (10, 20, 40 μM) for 4 h and then were exposed to 50 mM of glucose for 60 min. After that, the protein expression of P-p38, P-JNK, p38, and JNK was determined by western blot assay (**A**). The band intensity of these proteins was measured by Total lab TL120 and was utilized to analyze the level of protein expression by normalizing with the band intensity of β-actin (**B**). The values are demonstrated as the means ± SEM from three diverse experiments. ^#^*P* < 0.05 versus the control group and *P < 0.05 or **P < 0.01 versus the glucose-treated group.

As the previous experiment implied that the activation of p38 and JNK were involved in high glucose-induced microglial activation, we investigated whether these signaling pathways are linked to the inhibitory effect of sesamin on BV2 microglial activation stimulated by high glucose. The results revealed that while high glucose considerably enhanced the phosphorylation of p38 and JNK compared to control, sesamin pretreatment in BV2 cells significantly deterred that enhancement in a dose dependent manner (Fig. 7A,B). These evidences hinted that anti-microglial activation effects of sesamin were related to the inhibition of p38 and JNK activation.

### Inhibitory effects of sesamin on high glucose-induced microglial activation defended PC12 cells from neurotoxicity triggered by activated microglia

**Figure 8:**
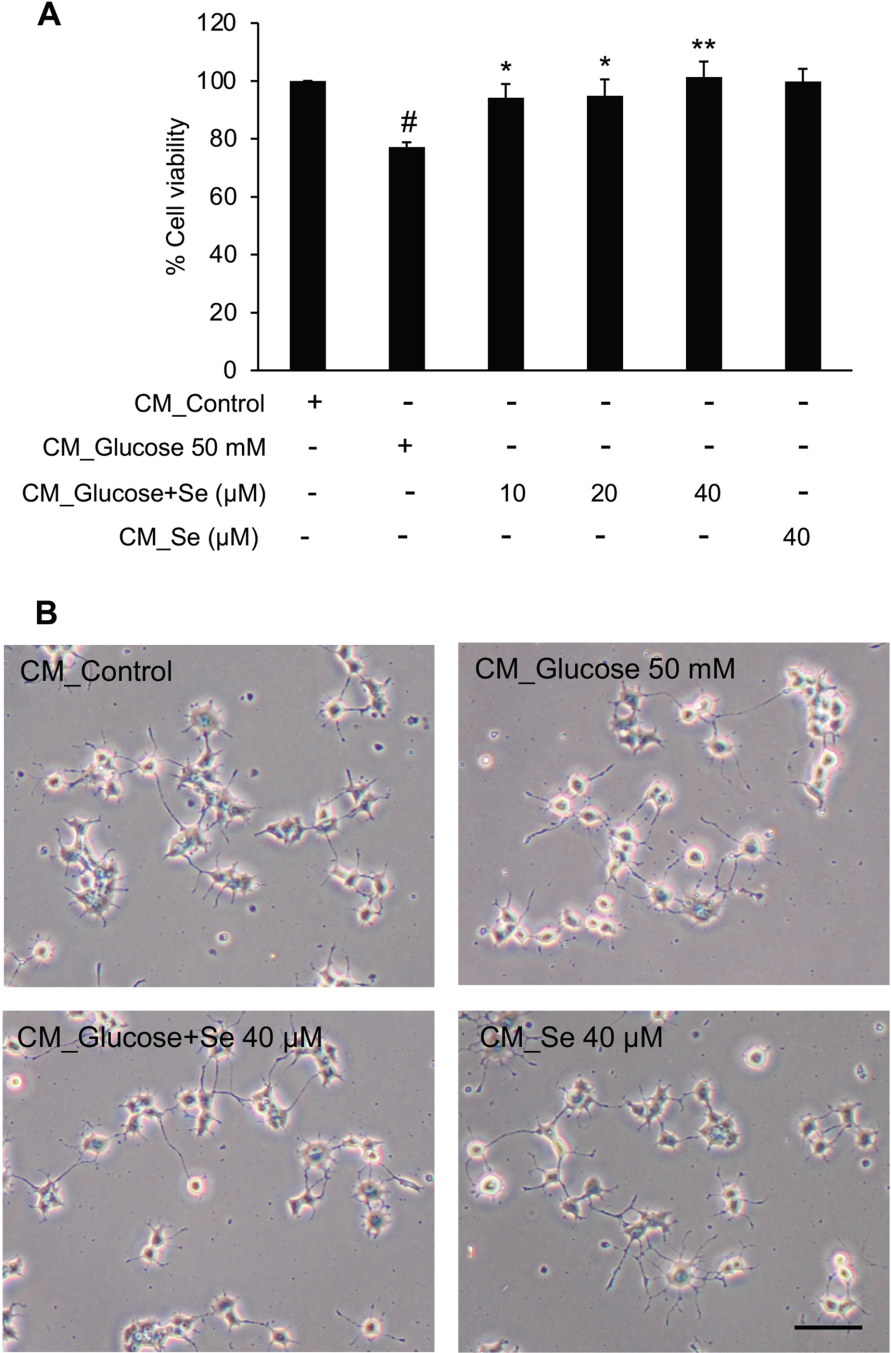
Indirect effects of sesamin on PC12 cell neurotoxicity induced by conditioned medium (CM) from high glucose-activated BV2 microglia. PC12 cells were exposed to CM from the BV2 cell culture. CM collected from BV2 cells were divided into six groups: CM from control BV2 cells (CM_Control), CM from BV cells treated with glucose alone (50 mM) (CM_Glucose 50 mM), CM from BV cells treated with sesamin (10, 20 and 40 μM) and glucose (50 mM)- (CM_Glucose + Se), and CM from BV cells treated with sesamin (40 μM) alone (CM_Se). Following incubation with CM for 48 h, PC12 cell viability was detected by MTT assay (**A**), and the morphology of PC12 cells was observed using a light microscope (**B**). In morphological observation, photos were taken at ×20 magnification and selected from one experiment as a representative from three independent experiments. The scale bar is 100 μm. The data is presented as a mean ± SEM of three different experiments. ^#^*P* < 0.05 versus CM_Control group and *P < 0.05 and **P < 0.01 versus the CM_Glucose 50 mM group.

The model of culturing differentiated PC12 cells in BV2 microglial conditioned medium (CM) has been vastly used in previous researches to study whether the anti-microglial activation effect of compounds result in the protection of neurons against neuronal damage or death provoked by microglial activation^40^. In this experiment, we investigated the neuroprotective effect of sesamin by treating PC12 cells with the conditioned medium collected from BV2 cell culture for 48 h. The neurotoxicity and morphology of PC12 cells were then determined using MTT assay and light microscope, respectively. The results showed that CM from BV2 cells treated with high glucose-alone caused a significant decrease in PC12 cell viability comparing with the CM from control BV2 cells, implying that it had a neurotoxic effect on PC12 cells. In contrast, the cell viability was not diminished in PC12 cells incubated with CM from high glucose and sesamin treated BV2 cells (Fig. 8). Corresponding to the neurotoxicity results, the detrimental morphology with a smaller soma and degraded neurite outgrowth was found in PC12 cells cultured in the CM from high glucose-stimulated BV2 cells. As expected, the damaged cell morphology was improved in PC12 cells cultured in the CM from sesamin-pretreated BV2 cells. These results point out that anti-microglial activation capabilities of sesamin in BV2 cells could subsequently protect PC12 cells from activated microglial-induced neurotoxicity.

## Discussion

DM is one of the most common chronic metabolic disorders worldwide and the major characterization of DM is high blood glucose, hyperglycemia, which contributes to severe brain complications, such as NDDs^41,42^. Since many studies affirmed that type 2 DM patients have a higher risk of developing NDDs (AD and PD) when compared with non-diabetic patients^43,44^, the discovery of the preventive strategy for DM-induced NDDs has become critical for the diabetic patients.

Currently, growing evidence confirmed the crucial role of microglia in the development of diabetes neuropathies as well as NDDs^45–47^. While any brain injury or pathological stimuli can trigger activation of brain’s microglia, the counterpart of peripheral macrophages, which are serving as a sensor of brain injury, the activation leads to various inflammatory responses during which an abundance of inflammatory mediators and neurotoxic factors were excreted influencing neighboring neurons and brain homeostasis processes. Excessive microglial activation and subsequent neuroinflammation undoubtedly initiate deterioration of neurons resulting in progression of NDDs^18,48–50^. It has been confirmed that hyperglycemia in diabetic conditions can cause an inflammatory microglial activation, and consequent neurodegeneration in the distinct NDDs models like AD and PD^47,51–54^. From all evidence, we hypothesized that modulation of microglial activation may be a therapeutic option in diabetes-aggravated NDDs.

Although Hwang and research group reported that extent of intracerebral glucose increased upon hyperglycemia was smaller in type 2 DM patients compared with healthy volunteers^55^, other research revealed the concentration of brain glucose is escalated in chronically hyperglycemic diabetic rat^21,56^. Over the past few years, many studies have applied high dosage-glucose treatment to imitate the hyperglycemic condition of diabetes in in vitro experiments which are discovering the mechanism of brain complications triggered by hyperglycemic circumstances^57–59^. Our study, likewise, utilized high glucose doses to attest its effects on microglial cells; 12.5, 25, 50 mM glucose were used to mimic the sugar levels in diabetes mellitus and its complications, diabetic ketoacidosis and hyperglycemia hyperosmolar status^24,60^. We discovered that the high glucose concentrations could clearly promote the activation of microglia along with a significant increment in the production of inflammatory factors particularly pro-inflammatory cytokines (IL-1β and TNF-⍺) and nitric oxide (NO), and their productions were regulated at their mRNA level. In addition, we confirmed the role of high glucose in the microglial activation utilizing the morphology of BV2 cells which was altered from the resting ramified feature to the activated amoeboid feature after being exposed to high dosage glucose. Our results are consistent with prior studies that suggested high glucose could induce inflammatory microglial activation in vitro^23,59,61^.

Here, many previous studies implicating high glucose treatment proved that correction of osmolality showed no significant effect on inflammation or oxidative stress as the use of glucose itself in various neuronal cells^58,62–64^. While high glucose at 50 mM significantly augmented LPS-induced microglial activation reflected by increased production of cytokines such as TNF-α and IL-6, the osmotic control, mannitol, did not show similar changes in primary rat microglia cells^58^. Similarly, in BV2 cells, high glucose, 25 mM or 33 mM, significantly induced microglial activation and ROS production, but, mannitol, in the concentration comparable to the glucose used, did not^63,64^. Although there was no previous study that used osmolality correction as high as 50 mM in BV2 cells, one study in PC12 cells demonstrated that the influence of mannitol was not seen until the osmolality increased up to 150 mM^62^. Hence, it can be assured that change in osmolality due to high dose glucose used in this study does not interfere with the results on effects of high glucose, including inflammatory responses and oxidative stress, observed in BV2 cells.

MAPK signaling pathways have been informed to be important pathways involved in hyperglycemia or high glucose-persuaded microglial activation in diabetic models. Previous examinations revealed that high glucose caused the elevated phosphorylation of MAPKs, p38, JNK, and ERK, in microglia^22,23,65^. Consistent with these data, in this study, a significant increment of phosphorylated MAPKs, except ERK, was observed in BV2 cells treated with 50 mM glucose; the phosphorylation of ERK was slightly increased though not significant.

As mentioned above, limiting microglial activation may be the potential way to prevent diabetic NDDs. Over a decade, phytochemicals have received an increasing interest for their therapeutic effects on several diseases along with lesser adverse effects^66,67^. Therefore, we paid an attention to sesamin, a phytochemical with numerous reported beneficial effects on diabetes and its brain complications. Sesamin is a type of lignans extracted from sesame seed oil (*Sesamum indicum*) and its powerful antioxidant and anti-inflammatory properties were approved by various studies^28,68,69^. Since there are in vivo affirmations of the blood–brain barrier permeability of sesamin^70^, recent researches focused on the neuroprotective effects of this compound. Sesamin exhibited the inhibitory effects against microglial activation induced by lots of stimuli^71–73^ and also a protective role in many neurodegenerative models, for instance, AD and PD^27,74–76^. Furthermore, Farbood and associates presented that sesamin defended rats from diabetes-aggravated hyperglycemia and subsequent cognitive decline^34^. Although protective effects of sesamin against hyperglycemia-mediated neuropathies have been confirmed, its effects on neurotoxicity, triggered by hyperglycemia via microglial activation, have not been clarified yet. Hence, we tried to find out whether sesamin inhibits the microglial activation under high glucose condition and its subsequent neuroprotective effect.

From our results, sesamin was shown to hinder high glucose-caused microglial activation. In high glucose-exposed BV2 microglia, a decrease of activated amoeboid morphology was observed when pretreated with sesamin in addition to a significant reduction of the release of inflammatory mediators like nitric oxide, IL-1β, and TNF-⍺ which was regulated at the transcriptional level. Furthermore, the anti-microglial activation abilities of sesamin were found to be related with the decrement of p38 and JNK phosphorylation corresponding with previous studies which proved that the inhibitory effects of sesamin against microglial activation are linked with the restraint of p38 and JNK signaling pathways^72,73,77^. Overall, we could imply that sesamin diminished inflammatory microglial activation under high glucose condition through inhibition of the p38 and JNK activation.

The system of culturing differentiated PC12 cells in BV2 microglial-conditioned medium (CM), which can emulate the activated microglia-neurons interaction in brain, has been utilized in the research that are interested in the effects of compounds on neurodegeneration triggered by activated microglia^40,78,79^. We applied this system to investigate how sesamin affects microglial activation-induced neuronal detriment in this study. While the CM from high glucose-treated BV2 cells had a toxic effect on differentiated PC12 cells as expected, the pre-incubation of BV2 cells with sesamin could defend PC12 cells from neurotoxicity, interestingly. Both cell viability and morphology of PC12 cells exposed to the CM from activated BV12 cells were improved by the pretreatment with sesamin. It is suggestive that sesamin protects PC12 cells by downregulating the production and secretion of pro-inflammatory mediators, TNF-⍺, IL-1β and nitric oxide, from high glucose-stimulated BV2 cells.

However, blockade of production of harmful mediators from microglial activation is not the sole pathway of neuroprotection by sesamin. Sesamin itself also has a direct protective effect on neuronal cells since it was reported to inhibit the apoptotic cell death of PC12 cells resulting from the inflammation, oxidative stress or nitrosative stress^80,81^. In our previous work, the apoptosis of PC12 cells exposed to the conditioned medium from LPS-treated BV2 cells can be reduced by direct sesamin treatment on PC12 cells^81^. Moreover, Bournival et al. showed that sesamin protects PC12 cells from high glucose-induced cell death through anti-apoptotic properties or anti-oxidative effect via increasing superoxide dismutase activity^80^. These studies suggested sesamin might directly protects PC12 cells exposed to the CM from high glucose-activated BV2 cells. Approximately 20% of sesamin was remained in the media of BV2 culture after 24- or 48-h treatment period compared to the sesamin control at ‘0’ hour (Supplementary Fig. S1). Since there was still sesamin left in the conditioned media that was used to treat PC12 cells, this could be an additional protective factor acting directly in PC12 cells beside its inhibitory effect on BV2 cells activation. Regretfully, it was one of the limitations of this study not to explore the direct effect of sesamin in PC12 cells. Anyhow, at least from the results of previous studies, it can be speculated that sesamin act not only in BV2 cells but also in PC12 cells, directly protecting them probably through inhibition of apoptotic pathways via reduction of oxidative or nitrosative stress.

Altogether, our results suggested that sesamin could improve inflammatory microglial activation, under high glucose circumstance, resulting in the protection of PC12 cells against activated microglial-mediated neurotoxicity. These results pointed out that the suppression of microglial activation by sesamin may be a preventive approach for hyperglycemia-associated NDDs in DM patients. However, as this finding is from in vitro experiments, it is crucial to further affirm the role of sesamin as a protective factor against microglial activation-mediated neurodegeneration using the diabetic animal model and clinical trials.

## Methods

### Cell culture, treatment, and conditioned medium (CM) preparation

Murine BV2 microglial cell line was acquired from ICLC (Genova, Italy). These cells were cultured in low glucose (1 g/L or 5.5 mM) glucose Dulbecco’s modified Eagle’s medium (DMEM) (Gibco, Grand Island, NY, USA) supplemented with 10% (v/v) heat-inactivated fetal calf serum (FCS) (Gibco, Grand Island, NY, USA), 100 units/mL penicillin, and 100 μg/mL streptomycin (Gibco, Grand Island, NY, USA). Cell lines were sustained in 37 °C humidified incubator under 5% CO_2_. In the experiments, BV2 cells were seeded in different multi-well plates. The number of cells was 1 × 10^4^ or 5 × 10^4^ cells/well in 96- or 24-well plates, respectively. Next, these cells were untreated or pretreated with sesamin at concentrations of 10, 20, and 40 μM for 4 h and then treated with either various concentrations (12.5, 25, 50 mM) of glucose (Sigma-Aldrich, St. Louis, MO, USA) or only glucose at 50 mM for various durations such as 15–60 min or 24- or 48-h depending on the experiment.

The conditioned medium (CM) from BV2 cell culture was prepared to treat PC12 cells. CM was collected from 6 groups, comprising control, glucose 50 mM, glucose with sesamin (Se) 10 μM, glucose with Se 20 μM, glucose with Se 40 μM, and Se alone. After 48 h-exposure of BV2 cells to 50 mM glucose, the culture medium was gathered and centrifuged at 150*g* for 5 min prior to displacing cell debris. The CM was stored at − 20 °C and preheated before utilization. Then, CM was added to differentiated PC12 cell culture.

Rat pheochromocytoma PC12 cell line was procured from CLS (Eppelheim Germany). The cells were grown in 10% (v/v) horse serum, HS (Gibco-BRL, Grand Island, NY, USA), 5% (v/v) FBS, 100 U/mL penicillin, and 100 μg/mL streptomycin mixed in high glucose (4.5 g/L or 25 mM) DMEM. Before plating PC12 cells in multi-well plates, 0.1 mg/mL poly-l-lysine (Sigma-Aldrich, St. Louis, MO, USA) was coated on each well plate. Cell concentration was 2 × 10^4^ or 4.5 × 10^5^ cells/well in 96- or 6-well plates, respectively. The experiment was performed after the induction of PC12 cells by 40 ng/mL of nerve growth factor (NGF, R&D Systems, Minneapolis, MN) to be differentiated into neuron-like cells.

### Preparation of sesamin

Sesamin was extracted from sesame seeds obtained from Lampang province, Thailand. The voucher specimens (BKF no. 138181) were approved by the National Park, Wildlife and Plant Conservation Department, Ministry of Natural Resources and Environment, Bangkok, Thailand. Sesamin was prepared as previously reported in Phitak’s study and confirmed to be identical to trustworthy sesamin sources (Sigma Aldrich)^82^. Sesamin obtained was firstly dissolved in dimethyl sulfoxide (DMSO) to make a stock solution (100 mM) and then diluted to desired concentrations using the culture media. All the experiments are done in compliance with relevant institutional guidelines.

### Cytotoxicity assays

The cytotoxicity of glucose or sesamin in BV2 cells was investigated by 3-(4,5-dimethyl-thiazol-2-yl)-2, 5-diphenyltetrazolium bromide, MTT (Sigma-Aldrich, St. Louis, MO) assay. Concisely, BV2 cells were treated with several concentrations of glucose or sesamin for 48 h. For PC12 cells, they were treated with CM from different groups. After the treatment, both BV2 and PC12 cells were incubated with 0.5 mg/mL MTT solution diluted in DMEM without serum for 4 h under 37 °C. Next, the DMEM was replaced by dimethyl sulfoxide (DMSO) (Sigma-Aldrich, St. Louis, MO, USA) to dissolve the formazan crystals. After shaking the plate, the optical density (OD) at 540 nm was measured using a microplate reader. The results were shown as the percentage of cell viability compared with the control group.

### Morphological examination

The morphology of BV2 cells and PC12 cells were inspected under a light microscope and pictures were taken at 20× magnification using EOS Utility Software—Canon USA, Inc. In BV2 cells, we observed the transformation from resting state (ramified shape) to activated state (amoeboid shape). In PC12 cells, we observed the appearances of neurodegeneration.

### Real-time PCR assay

The extraction of total cellular RNA from BV2 cells was carried out using the illustra RNAspin Mini Kit (GE healthcare, Europe GmbH, Freiburg, Germany). In reverse transcription processes, 500 ng of total RNA was converted to cDNA using iScriptTM cDNA synthesis kit (Bio-rad, Hercules, CA, USA) according to the provider’s protocol. The mRNA levels of inflammatory genes were determined by real-time PCR using SensiFAST TM SYBR^®^ Lo-ROX (Bio-Rad, Hercules, CA, USA). Reactions were directed in Applied Biosystems 7500/7500 Fast Real-Time PCR system. The mRNA levels of target genes were estimated relatively with that of β-actin (*ACTB*) using the 2^−ΔΔC(T)^ method. The primer sequences of genes used in this study are described in Table 1.

**Table 1:** List of primer sequences used for the mRNA level study.

Gene names	Sequence (5′–3′)	Accession number
*IL1B*	F: GACCTGCTTCTTTGAGGCTGAC R: TTCATCTCGAAGCCTGCAGTG	NM_031512.2
*IL6*	F: AAAGAGTTGTGCAATGGCAATTCT R: AAGTGCATCATCGTTGTTCATACA	NM_012589.2
*TNFA*	F: CTCCAGCTGGAAGACTCCTCCCAG R: CCCGACTACGTGCTCCTCACC	NM_012675.3
*iNOS*	F: CGTGAAGAAAACCCCTTGTG R: CAAAATCTCTCCACTGCCCC	NM_010927.4
*ACTB*	F: GTAAAGACCTCTATGCCAACA R: GGACTCATCGTACTCCTGCT	MN_031144.3

### Inflammatory cytokine release assay

The medium retained from the BV2 cell culture was examined for the level of inflammatory cytokines, including IL-1β and IL-6, using ELISA kits (R&D Systems, Minneapolis, MN, USA) according to the manufacturer’s instructions. The concentration of each inflammatory cytokine was calculated by the calibration curve of known concentrations of the respective inflammatory cytokine.

### NO release assay

The culture medium of BV2 cells was collected for nitric oxide (NO) measurement using Griess’s method. This medium was fused with the same volume of the Griess reagent. After incubating in the dark for 15 min, the absorbance at 540 nm was evaluated using a microplate reader. NO level in the medium was calculated from the sodium nitrite calibration curve and then shown as the relative values compared with the control group.

### Intracellular ROS assay

ROS generation in BV2 cells was investigated by measuring the fluorescence intensity of cultures after the addition of cell-permeable H2DCFDA (2′,7′-dichlorofluorescein diacetate) (Sigma-Aldrich, St. Louis, MO, USA). When the indicated treatment period was completed, cells were washed one time with phosphate-buffered saline (PBS) and incubated with 10 μM H2DCFDA for 30 min at 37 °C. After the washing step, the fluorescence intensity of dichlorofluorescein (DCF), the altered form of H2DCFDA induced by ROS oxidation, was quantified using a SynergyTM H4 Hybrid Multi-Mode Microplate Reader with 485 nm excitation and 528 nm emission wavelengths. The results were presented relative to the control group.

### Western blot assay

BV2 cells were harvested and lysed with lysis buffer after being exposed to the intended treatments. Cell debris was removed following the centrifugation, and then the supernatants were collected. The Bradford protein reagent (Bio-Rad, Munich, Germany) was used to measure protein concentrations. The separation of proteins was executed using 12% SDS-PAGE before the proteins were shifted to the nitrocellulose membrane (GE Healthcare Europe GmbH, Freiburg, Germany). After that, the membranes were blocked and exposed to specific antibodies, antibodies against p38, p-p38, JNK, p-JNK, ERK, p- ERK, and β-actin overnight at 4 °C. The used dilution of these antibodies (Cell Signaling Technology, MA, USA) was 1:1000. The membranes were then incubated with horseradish peroxidase (HRP)-conjugated secondary antibody (1:1000 dilution) prior to the band detection using a Supersignal West Femto Maximum Sensitivity Substrate kit (Thermo Fisher, Lifetechnologies). The relative band intensity to control was analyzed by the TotalLab TL120 software v2006 (for Windows, TotalLab software, Newcastle upon Tyne, UK, www.nonlinear.com). β-actin was used as the internal control.

### Statistics

The experiments were repeated at least three times independently and the results were expressed as a mean ± SEM. The data from the experiment were analyzed using the one-way analysis of variance (ANOVA) followed by Post Hoc multiple comparison. The calculations were carried out using SPSS software. P-value < 0.05 or 0.01 indicated the statistical significance.

## Conclusion

The present results revealed that high glucose condition induced microglial activation and inflammatory reactions in BV2 cells via the activation of p38 and JNK signaling pathways. Additionally, the CM of high glucose-activated BV2 cell culture induced neurotoxicity in differentiated PC12 cells. This outcome indicated that neurotoxicity was mediated by high glucose-provoked microglial activation. Next, we discovered that sesamin possessed the potent abilities to restrain inflammatory microglial activation and the underlying mechanism correlated with these inhibitory potentials is the modulation of p38 and JNK activation. Moreover, the anti-microglial activation effects of sesamin could protect PC12 cells against activated microglia-promoted neurotoxicity. Thus, sesamin might be a promising compound to be used for the prevention of diabetes-associated NDDs.

## Supplementary Information

Supplementary Figures.

## Data Availability

The datasets used and/or analysed during the current study available from the corresponding author on reasonable request.
