# Supplementary material for: Sesamin protects against neurotoxicity via inhibition of microglial activation under high glucose circumstances through modulating p38 and JNK signaling pathways
Source: Sci Rep. 2022 Jul 4;12:11296. doi: 10.1038/s41598-022-15411-3 (PMC9253356; doi:10.1038/s41598-022-15411-3)
Supplement: Supplementary file 1 — Supplementary Figures. [file 41598_2022_15411_MOESM1_ESM.docx]

**Sesamin protects against neurotoxicity via inhibition of microglial activation under high glucose circumstances through modulating p38 and JNK signaling pathways**

Prachya Kongtawelert^1^*, Chayanut Kaewmool^1^, Thanyaluck Phitak^1^, Mattabhorn Phimphilai^2^, Peraphan Pothacharoen^1^, Thuzar Hla Shwe^1^

^1^Thailand Excellence Center for Tissue Engineering and Stem Cells, Department of Biochemistry, Faculty of Medicine, Chiang Mai University, Chiang Mai, Thailand. 50200.

^2^Division of Endocrinology, Department of Internal Medicine, Chiang Mai University, Chiang Mai, Thailand.

**Supplementary Figures**

**
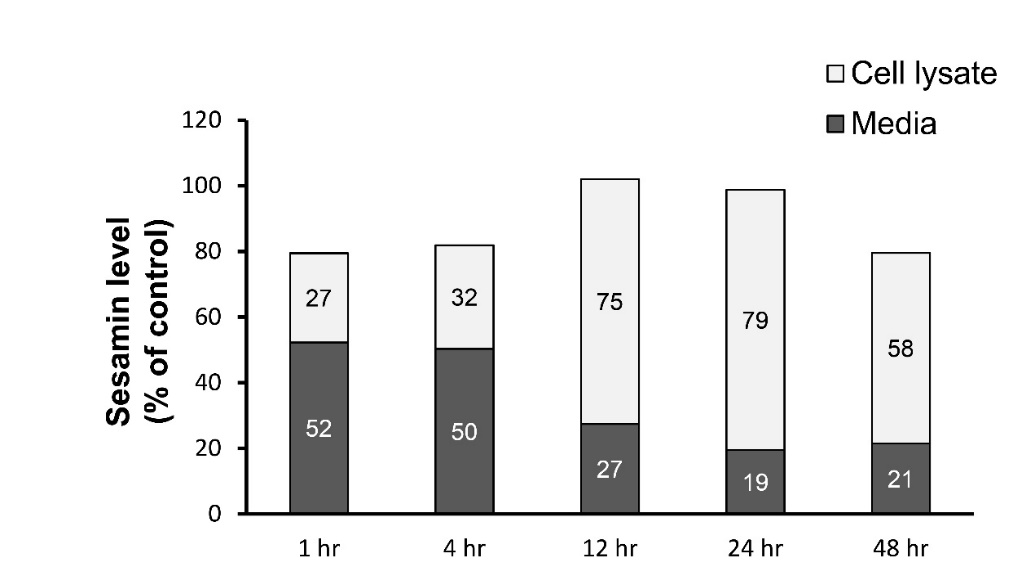
**

**Figure S1:** Sesamin level in media and cell lysate of BV2 cells. A total of 1x10^5^ cells/well of BV-2 were seeded in 6 well-plates. Cells were treated with sesamin at 50 µM. Media and cell lysate were collected at time periods of 1, 4, 12, 24 and 48 hours, and sesamin level was determined by high-performance liquid chromatography (HPLC). Sesamin level at each time period was calculated relative to sesamin control at ‘0’ hour.

**Supplementary figure for Figure 4**

**P-p38**


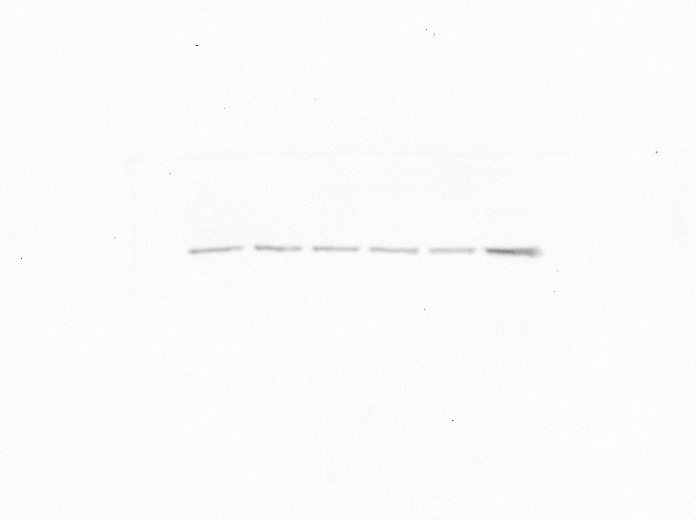


1 2 3 4 5 6


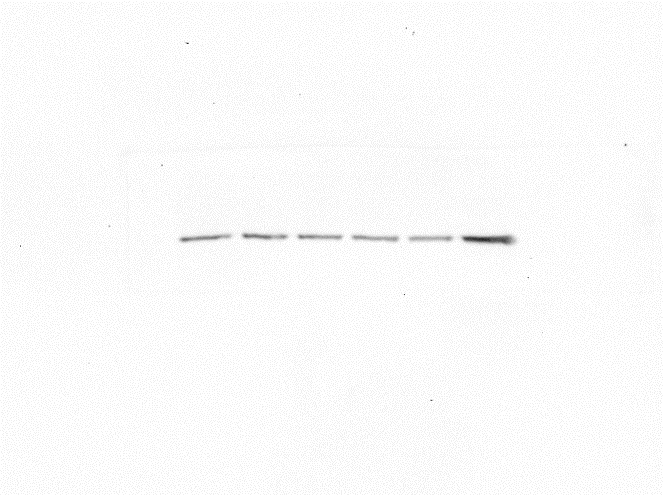


1 2 3 4 5 6

**Phospho-p38**

**(~ 40 kDa)**


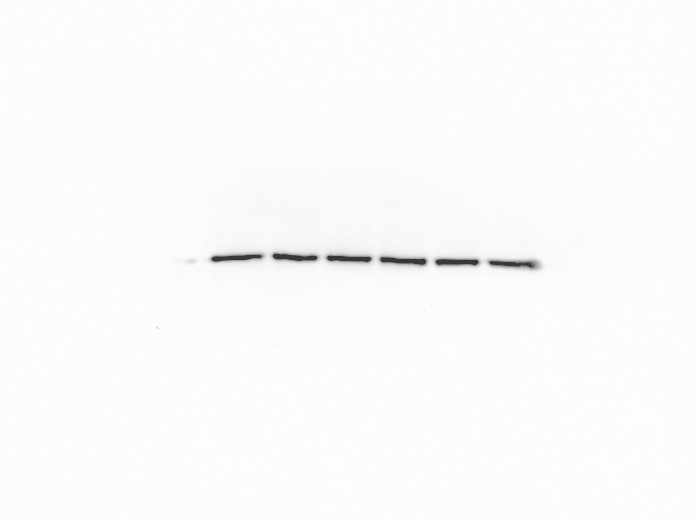
**p38**


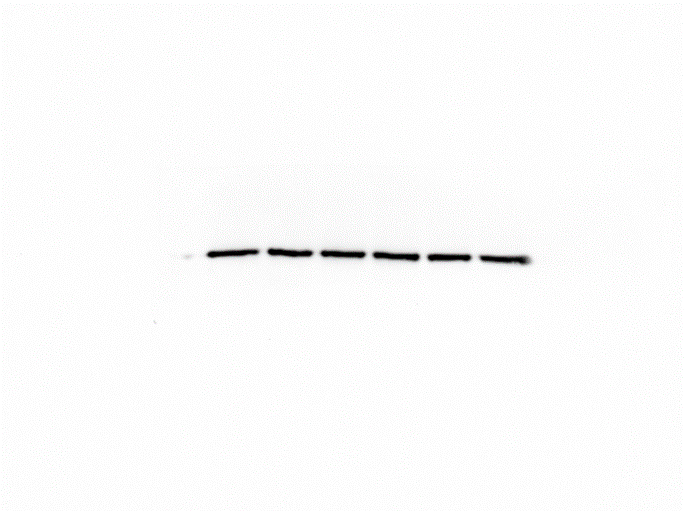


1 2 3 4 5 6

**Total-p38**

**(~ 40 kDa)**

1 2 3 4 5 6

**β-actin**


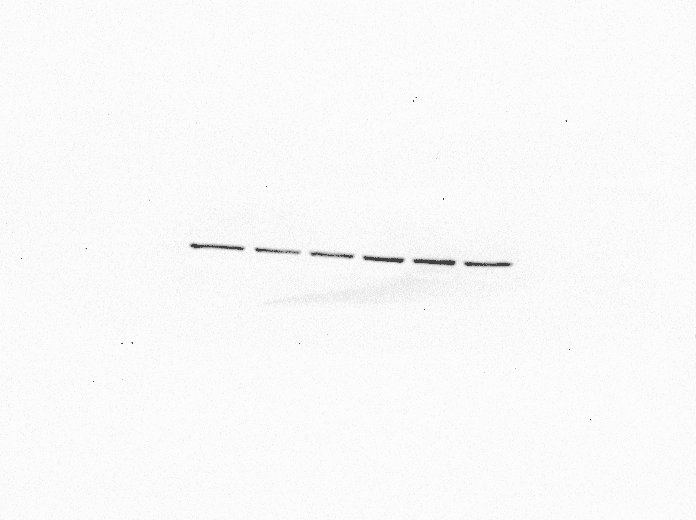
**
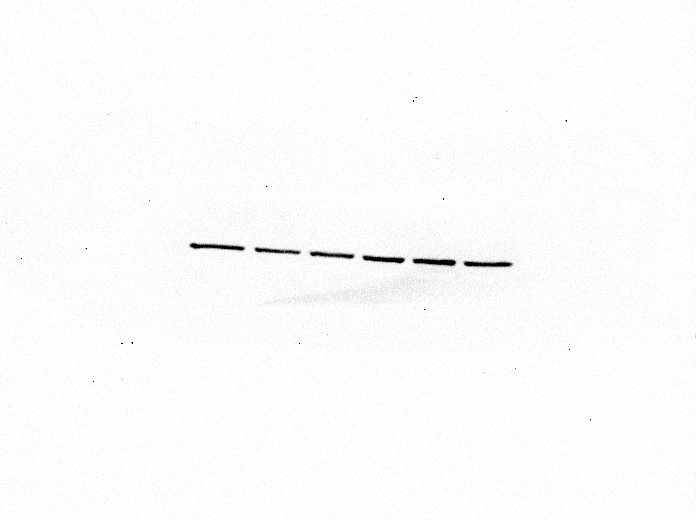
**

**β-actin**

**(~ 42 kDa)**

1 2 3 4 5 6

1 2 3 4 5 6

Bands

1 = Control: 15 min incubation

2 = Glucose 50 mM: 15 min incubation

3 = Control: 30 min incubation

4 = Glucose 50 mM: 30 min incubation

5 = Control: 60 min incubation

6 = Glucose 50 mM: 60 min incubation

**Supplementary figure for Figure 4 (Continue)**

**P-JNK**


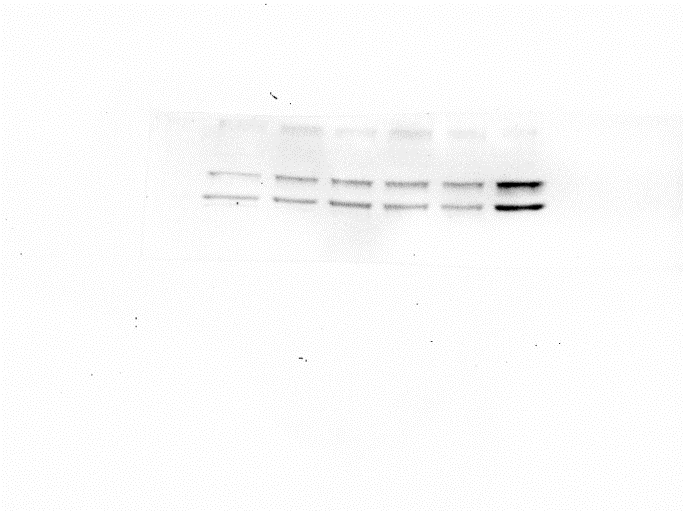


1 2 3 4 5 6


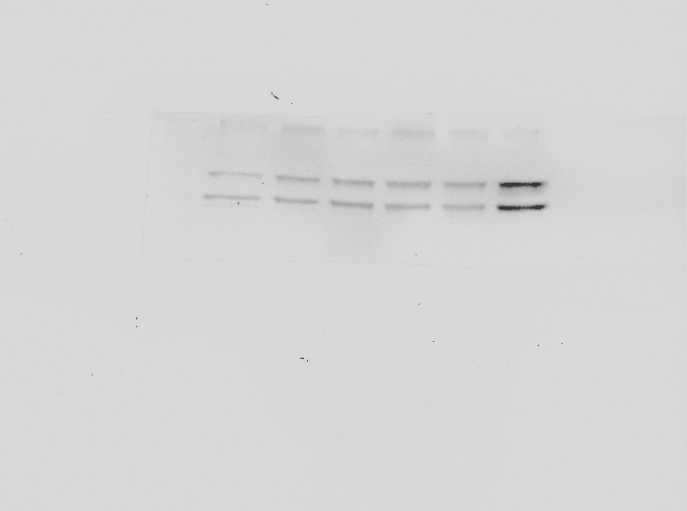


1 2 3 4 5 6

**Phospho-JNK**

**(~ 46, 54 kDa)**

**JNK**


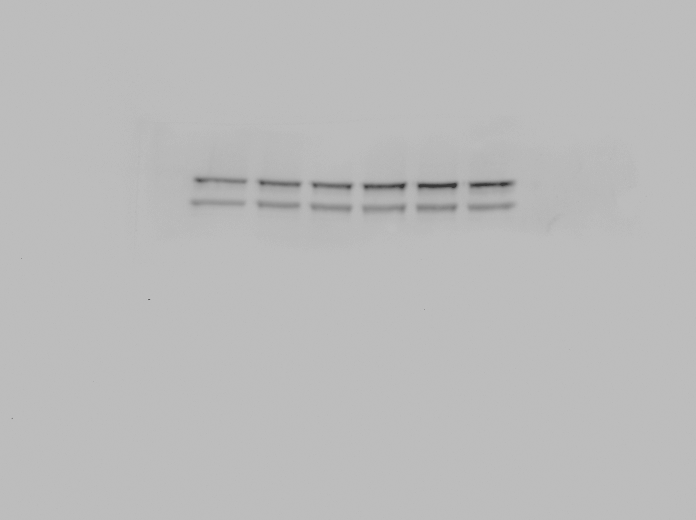


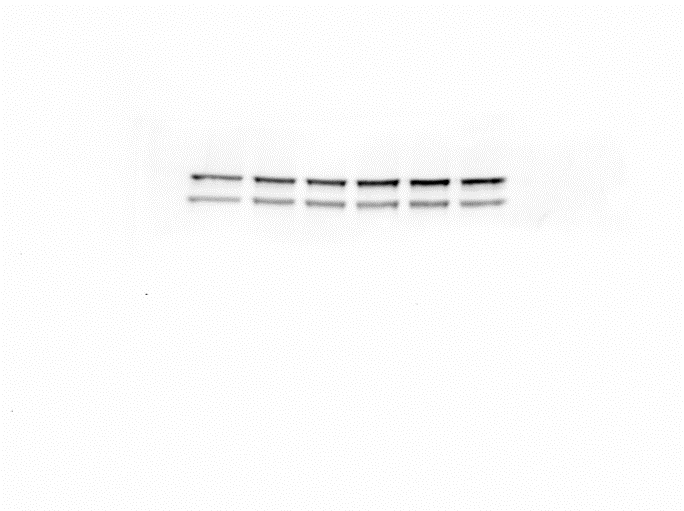


1 2 3 4 5 6

**Total-JNK**

**(~ 46, 54 kDa)**

1 2 3 4 5 6

**β-actin**


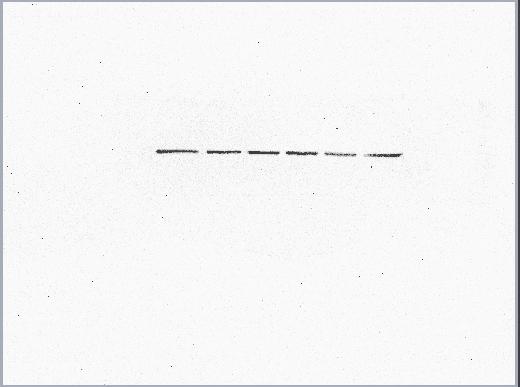


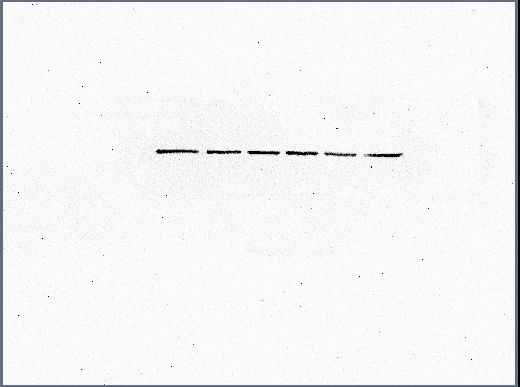


1 2 3 4 5 6

1 2 3 4 5 6

**β-actin**

**(~ 42 kDa)**

Bands

1 = Control: 15 min incubation

2 = Glucose 50 mM: 15 min incubation

3 = Control: 30 min incubation

4 = Glucose 50 mM: 30 min incubation

5 = Control: 60 min incubation

6 = Glucose 50 mM: 60 min incubation

**Supplementary figure for Figure 4 (Continue)**

**P-ERK**


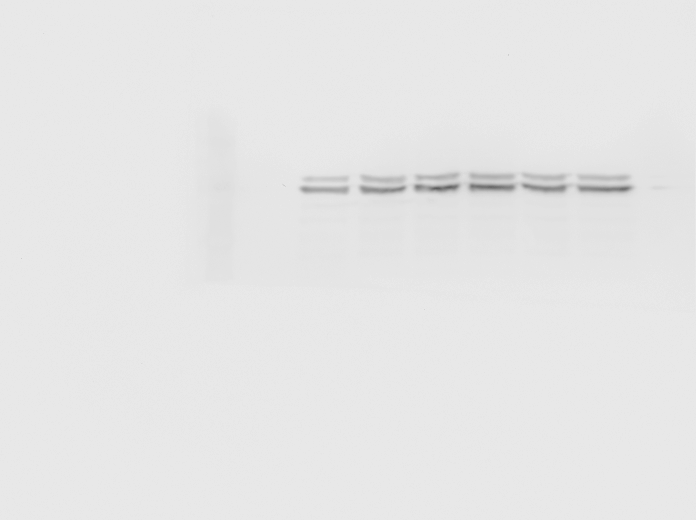


1 2 3 4 5 6


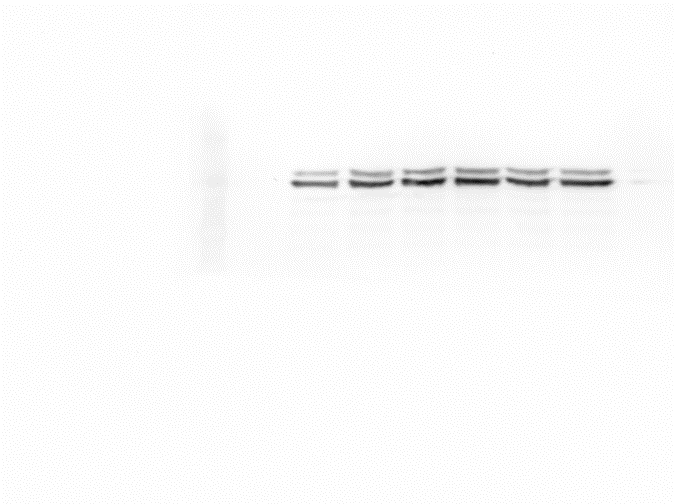


1 2 3 4 5 6

**Phospho-ERK**

**(~ 42,44 kDa)**

**ERK**


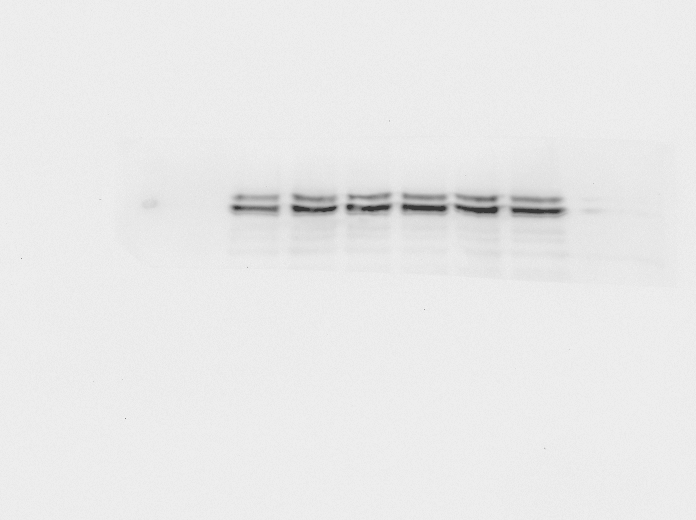


1 2 3 4 5 6


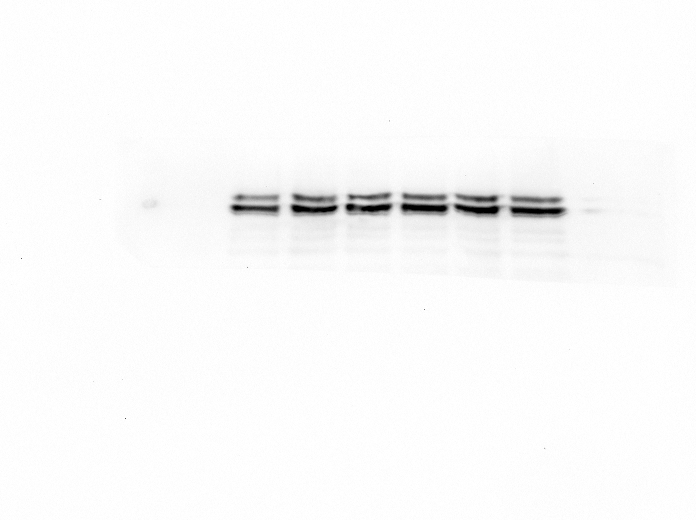


1 2 3 4 5 6

**Total-ERK**

**(~ 42,44 kDa)**


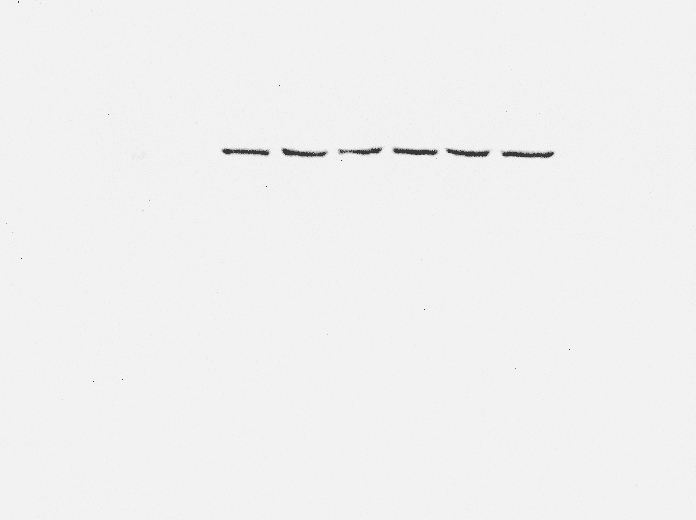
 **β-actin**


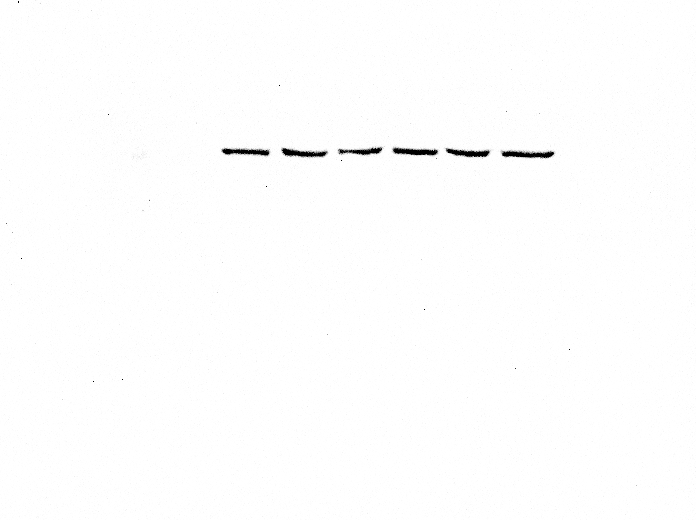


1 2 3 4 5 6

1 2 3 4 5 6

**β-actin**

**(~ 42 kDa)**

4 = Glucose 50 mM: 30 min incubation

5 = Control: 60 min incubation

6 = Glucose 50 mM: 60 min incubation

Bands

1 = Control: 15 min incubation

2 = Glucose 50 mM: 15 min incubation

3 = Control: 30 min incubation

**Figure S2.** Uncropped full-length pictures with different exposure of Western blotting membranes presented in Figure 4. Membranes were often cut to enable blotting for multiple antibodies.

**Supplementary figure for Figure 7**

**
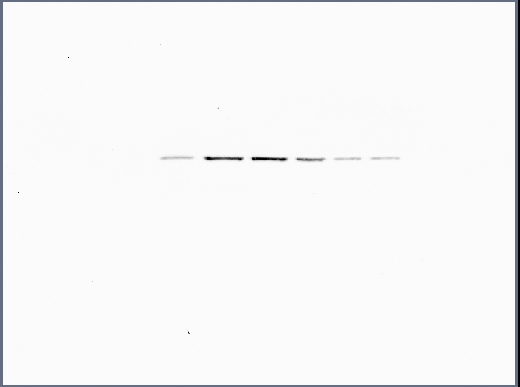
**
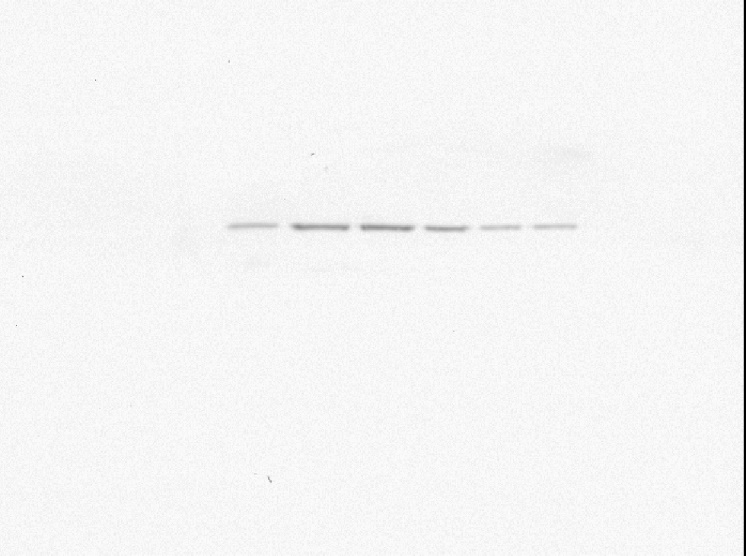
**P-p38**

**Phospho-p38**

**(~ 40 kDa)**

1 2 3 4 5 6

1 2 3 4 5 6

**p38**

**
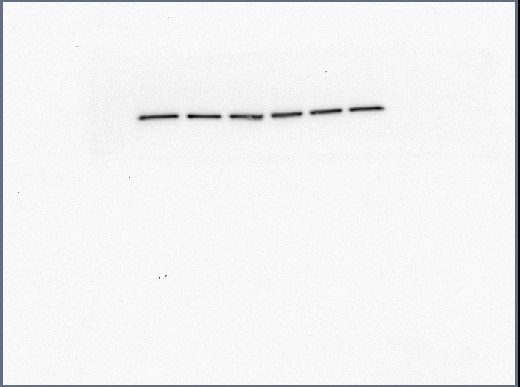
**


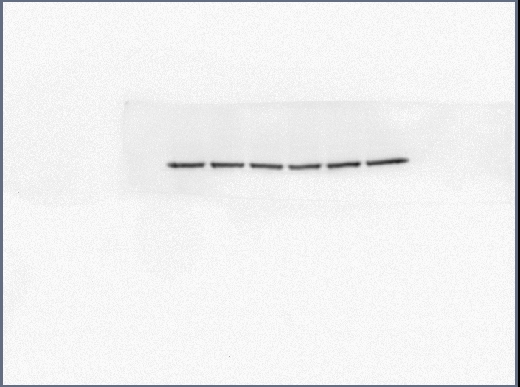


1 2 3 4 5 6

**Total-p38**

**(~ 40 kDa)**

1 2 3 4 5 6

**β-actin**

**
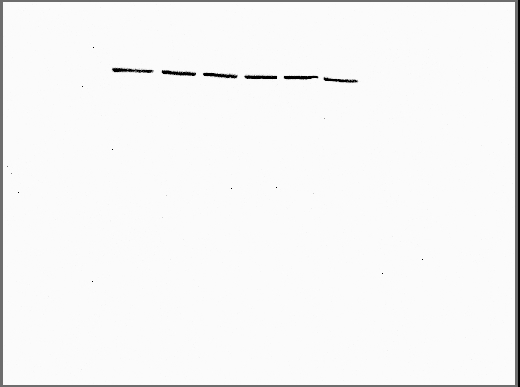
**


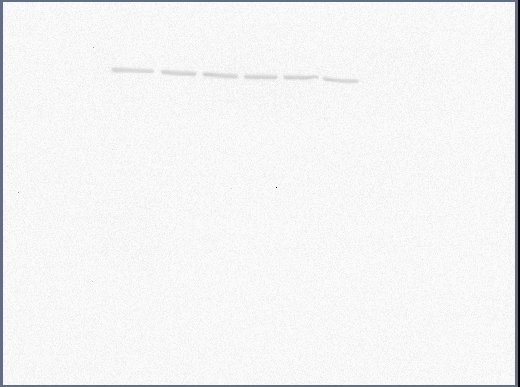


1 2 3 4 5 6

**β-actin**

**(~ 42 kDa)**

1 2 3 4 5 6

4 = Sesamin 20 uM + Glucose 50 mM

5 = Sesamin 40 uM + Glucose 50 mM

6 = Sesamin 40 uM

Bands

1 = Control

2 = Glucose 50 mM

3 = Sesamin 10 uM + Glucose 50 mM

**Supplementary figure for Figure 7 (Continue)**

**P-JNK**





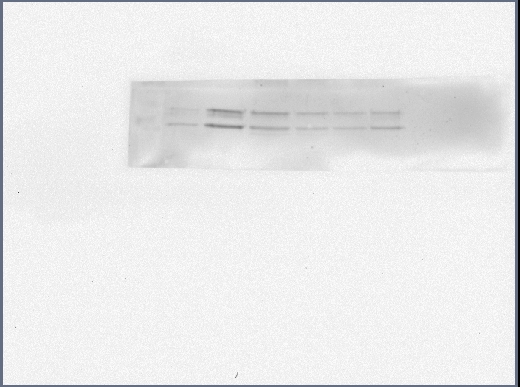


1 2 3 4 5 6

1 2 3 4 5 6

**Phospho-JNK**

**(~ 46, 54 kDa)**

**JNK**


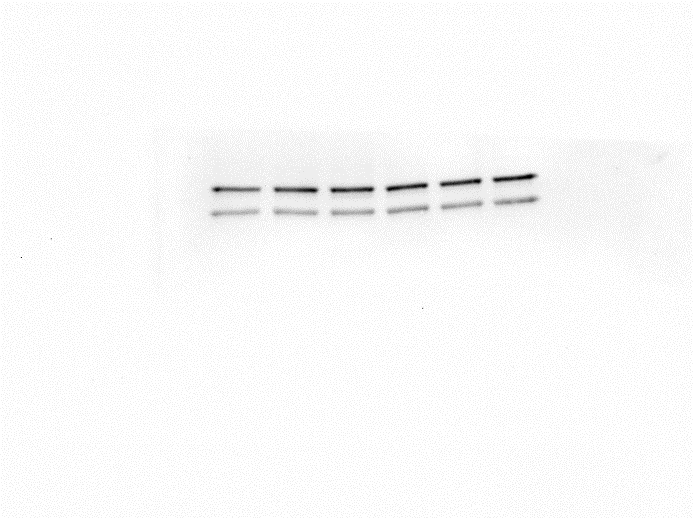


1 2 3 4 5 6


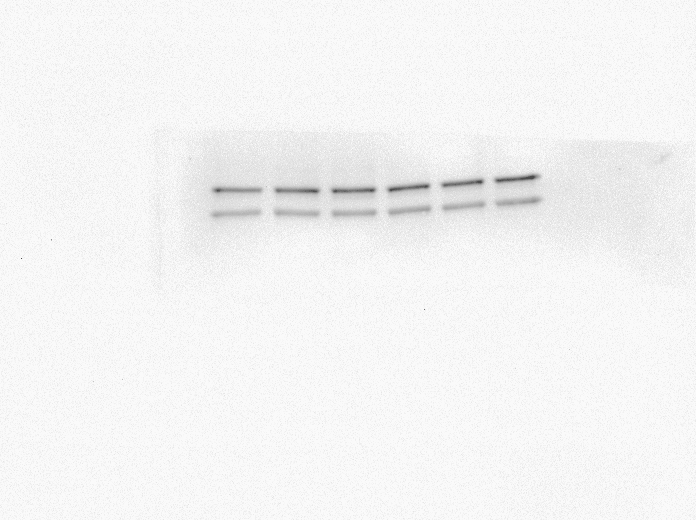


1 2 3 4 5 6

**Total-JNK**

**(~ 46, 54 kDa)**

**β-actin**

**
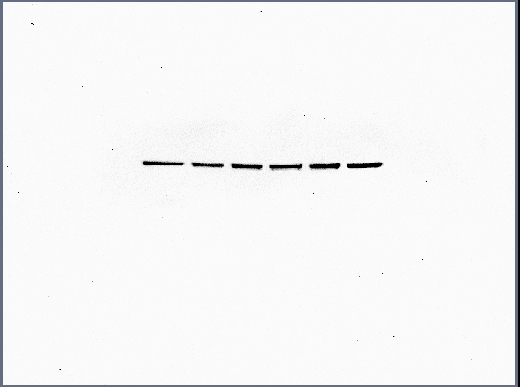
**


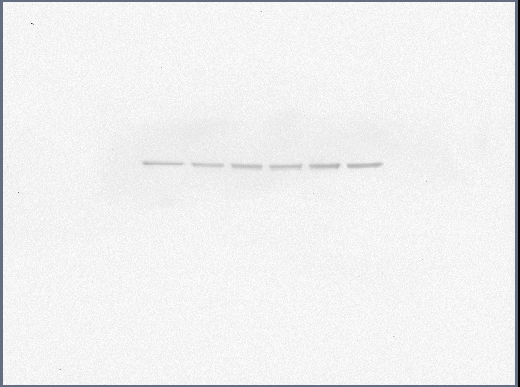


1 2 3 4 5 6

1 2 3 4 5 6

**β-actin**

**(~ 42 kDa)**

4 = Sesamin 20 uM + Glucose 50 mM

5 = Sesamin 40 uM + Glucose 50 mM

6 = Sesamin 40 uM

Bands

1 = Control

2 = Glucose 50 mM

3 = Sesamin 10 uM + Glucose 50 mM

**Figure S3.** Uncropped full-length pictures with different exposure of Western blotting membranes presented in Figure 7. Membranes were often cut to enable blotting for multiple antibodies.
